# Allele-Specific Gene Silencing in Two Mouse Models of Autosomal Dominant Skeletal Myopathy

**DOI:** 10.1371/journal.pone.0049757

**Published:** 2012-11-12

**Authors:** Ryan E. Loy, John D. Lueck, Mohammed A. Mostajo-Radji, Ellie M. Carrell, Robert T. Dirksen

**Affiliations:** Department of Pharmacology and Physiology, University of Rochester, Rochester, New York, United States of America; University of Queensland, Australia

## Abstract

We explored the potential of mutant allele-specific gene silencing (ASGS) in providing therapeutic benefit in two established mouse models of the autosomal dominantly-inherited muscle disorders, Malignant Hyperthermia (MH) and Central Core Disease (CCD). Candidate ASGS siRNAs were designed and validated for efficacy and specificity on ryanodine receptor (RyR1) cDNA mini-constructs expressed in HEK293 cells using RT-PCR- and confocal microscopy-based assays. *In vivo* delivery of the most efficacious identified siRNAs into flexor digitorum brevis (FDB) muscles was achieved by injection/electroporation of footpads of 4–6 month old heterozygous Ryr1^Y524S/+^ (YS/+) and Ryr1^I4895T/+^ (IT/+) knock-in mice, established mouse models of MH with cores and CCD, respectively. Treatment of IT/+ mice resulted in a modest rescue of deficits in the maximum rate (∼38% rescue) and magnitude (∼78%) of ligand-induced Ca^2+^ release that occurred in the absence of a change in the magnitude of electrically-evoked Ca^2+^ release. Compared to the difference between the caffeine sensitivity of Ca^2+^ release in FDB fibers from YS/+ and WT mice treated with SCR siRNA (EC_50_: 1.1 mM versus 4.4 mM, respectively), caffeine sensitivity was normalized in FDB fibers from YS/+ mice following 2 (EC_50_: 2.8 mM) and 4 week (EC_50_: 6.6 mM) treatment with YS allele-specific siRNA. Moreover, the temperature-dependent increase in resting Ca^2+^ observed in FDB fibers from YS/+ mice was normalized to WT levels after 2 weeks of treatment with YS allele-specific siRNA. As determined by quantitative real time PCR, the degree of functional rescue in YS/+ and IT/+ mice correlated well with the relative increase in fractional WT allele expression.

## Introduction

With the advent of high throughput sequencing and the completion of the human genome project, the implications of genetics on individualized patient health care are increasing exponentially. These advances open the door for a genetic basis of personalized medicine, enabling the possibility of tailoring individualized therapy based on the underlying genetics of the patient in addition to their particular symptomatic presentation. In this context, allele-specific gene silencing (ASGS), a strategy designed to selectively target and silence one allele at the mRNA level using RNA interference (RNAi), represents an attractive approach to correct the fundamental genetic defect in individuals suffering from autosomal dominant disorders [1].

Growing interest in the development and testing of RNA-based therapeutic strategies has been fueled by recent exciting clinical trial successes using small interference RNAs (siRNAs) to treat a wide variety of disorder including cancers, viruses and genetic disorders. For example, Macugen, a VEGF-specific modified RNA aptamer, has gained FDA approval for the treatment of age-related macular degeneration [2]. Early successes were also observed in clinical studies using oligonucleotide-based exon skipping strategies in autosomal recessive Duchenne muscular dystrophy [3], [4] and using pre-mRNA splice-altering morpholinos in mouse models of myotonic dystrophy [5], [6]. While RNAi knockdown strategies have shown promise for some dominantly inherited non-muscle disorders [1], this therapeutic approach has not yet been developed or tested for the treatment of autosomal dominant muscle diseases.

Central Core Disease (CCD) and Malignant Hyperthermia (MH) are autosomal dominant skeletal muscle disorders linked primarily to single amino acid substitutions in the sarcoplasmic reticulum Ca^2+^ release channel of skeletal muscle (the type 1 ryanodine receptor or RyR1). The recent generation of RyR1 knock-in mouse models for MH with central cores (Ryr1^Y524S/+^ or YS/+) [7] and CCD (Ryr1^I4895T/+^ or IT/+) [8] has provided tremendous insight into the pathomechanisms of these two diseases. YS/+ mice exhibit heat and halothane-induced sudden death, age-dependent formation of discrete regions of altered muscle ultrastructure or cores [9], and muscle fibers from YS/+ mice exhibit a marked sensitization to activation by halothane, caffeine, and voltage [7], [10]. IT/+ mice exhibit muscle weakness [11], [12], altered muscle ultrastructure [12], [13], and a reduction in RyR1 channel Ca^2+^ ion permeation that limits RyR1 Ca^2+^ release without changing caffeine or voltage sensitivity [11].

Given that MH and CCD are autosomal dominantly inherited skeletal muscle disorders and that ablation of one *Ryr1* allele is well-tolerated in mice [14], [15], we hypothesized that preferential ASGS of the mutant RyR1 allele in YS/+ and IT/+ knock-in mice would correct RyR1 function in these mice. Here we developed *in vitro* mRNA- and protein-based assays to screen multiple potential siRNAs for their ability to effectively and selectively silence the corresponding mutant RyR1 mRNAs. For the most promising of these siRNAs, we then evaluated the specificity and efficacy of these siRNAs in preferentially silencing the mutant allele and correcting RyR1 function following local *in vivo* delivery in footpad muscles of IT/+ and YS/+ mice. The results indicate that rescue of RyR1 function in each mouse model closely parallels the ability of the siRNA treatment to preferentially suppress mutant allele expression, and thus, to increase the relative proportion of WT allele expression. These findings provide “proof-of-principle” for the utility of siRNA-mediated ASGS as a potential therapeutic strategy to treat autosomal dominant RyR1-linked disorders.

## Materials and Methods

### Ethics Statement

The University of Rochester’s University Committee on Animal Resources (UCAR) reviewed and approved all the animal procedures performed in these studies (Protocol #2006-114R2001 ), which were aligned with the recommendations in the Guide for the Care and Use of Laboratory Animals of the National Institutes of Health. Electroporation of mouse footpads with siRNA was performed with mice under general anesthesia utilizing a mixture of ketamine, xylazine, and acepromazine and appropriate steps taken to minimize suffering.

### siRNA Design: Algorithm and Scoring

Dharmacon, Inc (Lafayette, CO) has determined eight characteristics for the proper design of successful siRNAs, which are incorporated into a rational siRNA design algorithm [16] available on their website (www.dharmacon.com). For allele-specific gene silencing of the RyR1 IT and YS mutant alleles, this scoring system was utilized to identify a series of siRNAs from potential sequence options around region of each mutation (Y522S in exon 14 and I4895T in exon 102). Using the Dharmacon algorithm, identified siRNAs were scored *in silico* for preferential binding and silencing of the mutant or WT sequence. The identified siRNAs and their corresponding scores are illustrated in Figure S1A and B.

#### Plasmid construction

Total RNA was isolated from the skeletal muscle of mice heterozygous for either the I4895T or Y522S RyR1 mutation using TRIzol® (Invitrogen, Carlsbad, CA) according to manufacturer’s instructions. Reverse transcriptional polymerase chain reaction (RT-PCR) using Superscript™ III First Strand Synthesis System for RT-PCR (Invitrogen, Carlsbad, CA) and Oligo(dT)_20_ priming was utilized to create total cDNA from which exons 100–105 of RyR1 for the IT mutation (exon 102) or exons 12–16 for the YS mutation (exon 14) were cloned into pcDNA3.1 flanked with NheI and MluI restriction sites. The WT or mutant exons were then transferred to one of two screening plasmids (venus for WT and cherry for mutant) via PCR amplification that created an MluI-flanked PCR product. The product was digested with MluI and ligated to MluI-linearized pCI-NEO-based vectors; WT sequences were inserted between Venus and a 3X FLAG epitope tag (Figure S1C and D, *left*). Mutant exon sequences were inserted between Cherry and a 3X HA epitope tag (Figure S1C and D, *right*). All inserts were fully sequenced to confirm directionality and sequence integrity.

#### Cell transfections

HEK293 (ATCC, Manassas, VA) cells were plated in 35 mm tissue culture dishes (Corning, NY) 24 hours before each transfection of cells at ∼60–70% confluence. Lipofectamine 2000 (Invitrogen, Carlsbad, CA) was used to simultaneously transfect the plasmids (3 µg of each plasmid) and the siRNAs (200 or 400 pmol each) according to Lipofectamine 2000 standard protocols with minor modifications [17].

#### RNA isolation

Total RNA was isolated 48 hours following transfection of HEK293 cells with siRNA screening plasmids and 200 pmol siRNA using TRIzol® (Invitrogen, Carlsbad, CA) according to manufacturer’s instructions. RNA concentration and purity were measured via absorbance using a NanoDrop-1000 (Thermo Scientific, Wilmington, DE). DNase I (Fermentas, Glen Burnie, MD) was used to remove any contaminating DNA.

#### Semi-Quantitative RT-PCR

**Figure 1:**
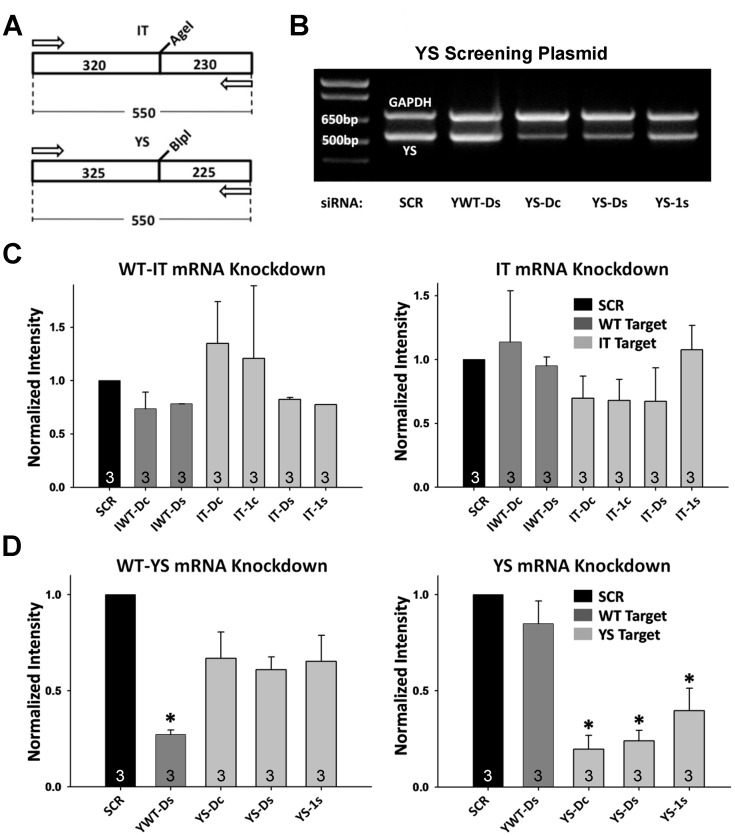
siRNA Screening Results at the RNA Level. A and B) Approach for screening siRNA knockdown efficiency and specificity by RT-PCR. A) Schematic of PCR design for IT (*left*) and YS (*right*) showing location of AgeI and BlpI restriction sites, respectively. B) Representative PCR products obtained using YS (*bottom band*) and GAPDH primers (*top band*) on mRNA obtained from HEK293 cells transfected with the YS screening plasmid. C and D) Average (±SEM) siRNA-mediated mRNA knockdown. Results are shown for both the WT (*left*) and mutant (*right*) screening constructs. C) IT-based screening and D) YS-based screening (* p<0.01 vs SCR control). n = 3 for all conditions.

First strand synthesis from total RNA was completed using Superscript™ III First Strand Synthesis System for RT-PCR (Invitrogen, Carlsbad, CA) and Oligo(dT)_20_ priming and RNA was removed using RNase H (Invitrogen, Carlsbad, CA). Efficiency of siRNA knockdown was assessed by PCR using target sequence primer pairs as depicted in Figure 1A and a second set of primers to amplify GAPDH for use as internal RNA concentration and loading controls. Cycle dependence of product accumulation was determined for all primer pairs and 26 cycles was chosen as the cycle number that fell within the exponential range of accumulation for all primer pairs (data not shown). After separation by standard agarose gel electrophoresis, products were imaged in an Epi Chemi II Darkroom and Labworks v 4.6 (UVP BioImaging Systems, Upland, CA) was used to analyze band intensity. Following background subtraction, IT or YS primer products were normalized to GAPDH products to control for loading. All results were normalized to scrambled (SCR) control siRNA.

#### Ratiometric confocal fluorescence microscopy

To assess siRNA protein knockdown specificity and efficacy under heterozygous expression conditions, equal amounts of venus-tagged WT and cherry-tagged mutant screening constructs (3 µg each) were transfected into HEK293 cells along with the designated siRNA as described above. Using this approach, a co-transfection efficiency for both fluorophores of <20%, ideal for isolated single cell imaging, was routinely achieved. Confocal fluorescence microscopy was used 2 days later to separately and sequentially image Venus (488 nm excitation) and Cherry (543 nm excitation) fluorophores. Identical settings for laser excitation intensity, dwell time (1.92 µs), pinhole diameter (30 µm), and photomultiplier sensitivity were used for each fluorophore across all experiments. The ratio of Venus (WT) to Cherry (mutant) fluorescence was determined offline using ImageJ (NIH, http://rsb.info.nih.gov/ij/). Results were normalized to scrambled (SCR) siRNA-transfected controls to compare across multiple transfections.

### Electroporation of Mouse Footpads for siRNA Delivery

siRNAs were delivered to the FDBs of anesthetized mice by injection and electroporation based on techniques previously described [18]. Briefly, 6 µl of 2 mg/ml hyaluronidase was prepared in PBS and injected subcutaneously into the hind foot of the animal using a 33-gauge needle. One hour later, 200 pmol of siRNA, dissolved in 1X siRNA buffer (in mM: 20 KCl, 6 HEPES-pH 7.5, and 0.2 MgCl_2_) supplemented with 71 mM NaCl, was injected. After 5 min, two electrodes (200 µm gold plated stainless steel needles) were placed subcutaneously near the proximal and distal tendons of the FDB muscles in order to deliver electrical pulses. The pulse protocol consisted of 20 pulses of 100 V for 20 ms duration at a frequency of 1 Hz. Contralateral FDB muscles received 200 pmol of SCR siRNA and served as controls. For 2-week treatment groups, 200 pmol of siRNA was injected and electroporated on day 0 and day 7; experiments were performed on day 14. For 4-week treatment groups, 200 pmol of siRNA was injected and electroporated on days 0, 7, 14, and 21; experiments were performed on day 28.

### Preparation of FDB Fibers

Flexor Digitorum Brevis (FDB) muscle fibers were isolated as described previously [19]. Briefly, FDB muscles were removed from the hind paws and cleaned of associated connective tissue while bathed in a control Ringer’s solution consisting of (in mM): 145 NaCl, 5 KCl, 2 CaCl2, 1 MgCl2, 10 HEPES, pH 7.4. Muscles were enzymatically dissociated in Ringer’s solution supplemented with 1 mg/mL collagenase A for 60 min at 37°C in a shaking incubator at 40 oscillations/minute. Mechanical dissociation by trituration completed final dispersal and plating of individual FDB fibers onto glass coverslips. Fibers chosen for experimentation possessed a clean morphology, striations, and no signs of swelling or damage. All experiments were conducted within 8 hours of fiber isolation.

### Semi-Quantitative RT-PCR from FDB Fibers

In order to determine allele frequency in scrambled-treated and mutant-targeted siRNA-treated fibers, total RNA was isolated from muscle fibers remaining attached to the tendon following trituration using RNA Easy (Qiagen, Valencia, CA) with the following modifications: fibers were centrifuged at 3000 × g in a benchtop centrifuge for 5 min at room temperature in Ringer’s solution. Fibers were resuspended according Qiagen RNA Easy instructions, aggressively passed through a 21 gauge needed 10–15 times, and then RNA preparation completed with a final elution volume of 30 µL. Two sets of primers, one amplifying the dihydropyridine receptor and the second amplifying RyR1, were used in a Qiagen’s OneStep RT-PCR system to perform RT-PCR on small quantities of RNA prepared from isolated FDB fibers to generate two PCR products. The products were digested overnight at 37^o^C in the presence of 10 U of T7 Endonuclease I (New England Biolabs, Ipswich, MA) to remove heteroduplexes and either the AgeI (to digest product originating from the IT allele) or BlpI (to digest product originating from the YS allele). The remaining undigested product reflects priming from the WT allele. Gel densitometry of the uncut PCR product before and after digestion was used to calculate the fraction of WT product. Loading and RNA variability was controlled for by normalizing to the dihydropyridine receptor PCR product, which was free of either the AgeI (IT) or BlpI (YS) restriction sites.

### Quantitative Real Time PCR (qRT-PCR) from Isolated FDB Fibers

Mouse footpads were electroporated with either SCR or mutant allele-targeted siRNAs for two weeks and then RNA was prepared from isolated FDB fibers as described above. First strand synthesis was completed using Superscript™ III First Strand Synthesis System for RT-PCR (Invitrogen, Carlsbad, CA). Following first strand synthesis, total cDNA was used as a template for quantitative real time PCR (qRT-PCR) using iTaq SYBR Green Supermix with ROX (Bio-Rad, Hercules, CA) using an ABI Prism 7000 Real-Time PCR Machine (Applied Biosystems, Carlsbad, CA). Three reactions were performed utilizing three different primer sets: 1) amplification of calsequestrin type1 was completed as a reference muscle-specific gene, 2) amplification of WT RyR1 allele using WT-specific primers, and 3) amplification of the appropriate mutant RyR1 allele using (YS or IT) using mutant-specific primers. Design of the allele-specific primers was similar to that used by Zvaritch et al. [12] and allele specificity was confirmed prior to qRT-PCR in high cycle number PCR reactions in which WT primer sets yielded no product with cDNA generated from homozygous YS and IT mice and mutant primer sets yielded no product from cDNA prepared from WT mice. All primer pairs were designed to amplify a product across multiple introns and reverse transcriptase negative controls were included to further rule out genomic DNA contamination. Dissociation curves were evaluated to confirm the presence of a single PCR product prior to analysis. Data were quantified using the ΔΔCt method to determine the fold change between WT and mutant alleles (an equal quantity of template results in a value of 1). For each muscle, reactions were carried out in quadruplicate. Data reported reflect the average (± SEM) of 4 muscles for each condition.

### Indo-1 Ca^2+^ Fluorometry

Single FDB fibers were loaded with 6 µM indo-1 acetomethoxy ester in Ringer solution for 30 min at room temperature. Fibers were then rinsed dye-free Ringer’s supplemented with 25 µM 4-methyl-N-(phenylmethyl) benzenesulfonamide (BTS, Tocris Biosciences, Minneapolis, MN), a skeletal muscle myosin II ATPase inhibitor, and incubated for >20 min to allow for de-esterification of the dye and inhibition of contraction. Cytosolic dye was excited at 350 nm using a 75 W xenon bulb and a high-speed DeltaRAM illuminator (Photon Technology International, Princeton, NJ). Fluorescence emission at 405 nm (F_405_) and 485 nm (F_485_) was collected (100 Hz) within a small rectangular region of the fiber using a 40X (1.35NA) oil-immersion fluorescence objective and a photomultiplier detection system (Photon Technology International, Princeton, NJ) with results presented as the ratio of F_405_ and F_485_ (R = F_405_/F_485_). Electrically-evoked Ca^2+^ transients were elicited using an electrical stimulus (8 V at 0.1 Hz) delivered using two extracellular electrodes filled with 1% agarose in 200 mM NaCl placed on either side of the cell of interest. Agonist-induced RyR1 Ca^2+^ release was triggered by rapid local application of 500 µM 4-choloro-*m*-cresol (4-CMC) or varying concentrations of caffeine. The maximum rate of 4-CMC-induced Ca^2+^ release was approximated from the peak of the first derivative of the indo-1 ratio (dR/dt). Caffeine concentration-response curves (0.1–60 mM) were applied directly to indo-1 loaded FDB fibers using a rapid perfusion system that sequentially exposed fibers to 30 s applications of increasing concentrations of caffeine (0.1, 0.3, 0.6, 0.8, 1.0, 3.0, 10.0, 30.0, and 60.0 mM) with each concentration followed by a 45 s wash with control Ringer’s solution. Data were plotted as the percent of all fibers responding (greater than 0.1 F_405_/F_485_ increase from baseline) to a given concentration of caffeine and fit with a 3-parameter Hill Equation to determine Hill coefficient (h) and EC_50_ values.

### Temperature Dependence of Resting Calcium

FDB fibers were loaded with 5 µM fura-2 AM (TEFLABS, Austin, TX) for 30 min at room temperature in control Ringer’s solution and rinsed dye-free Ringer’s supplemented with 25 µM BTS. Fura-2 was alternately excited at 340 nm and 380 nm using a Polychrome V monochromator-based illumination system (TILL Photonics, Munich, Germany). Images were captured (510 nm emission) using a high speed, digital SENSICAM-QE CCD camera (Cooke, Romulus, MI) and TILL Vision software at 25^o^C and 32^o^C controlled by a Dagan HW-30 Temperature Controller (Dagan, Minneapolis, MN). Ratio images (R = F_340_/F_380_) were generated using TILL Vision software and mean resting values calculated using ImageJ (NIH) for data recorded at 25^o^C and 32^o^C. For each condition, resting ratio images were first collected from multiple fibers in the dish while the temperature was held at 25^o^C. The temperature of the bath was then equilibrated to 32^o^C and additional ratio images were then imaged. This approach minimized deleterious effects of repeated heat/cool cycles, maximized the number of fibers imaged per dish, and minimized the number of animals needed.

### Statistics

All data are presented as mean ± SE. A one-way ANOVA and *post hoc* Holm-Sidak test was to evaluate statistical significance across multiple groups. An unpaired Student’s t-test was used to evaluate statistical significance where only two groups are compared.

## Results

### siRNA Design

Figures S1A and B define the siRNAs screened in this study. These siRNAs were predicted *in silico* to have the highest potential for effective and selective silencing of the mutant IT and YS alleles. The siRNAs were designed to possess either centrally positioned mismatches or shifted mismatches located between antisense bases 2–8. The siRNA nomenclature used throughout this study (e.g. YS-Dc) is a 2–3 letter genotype match (YS, IT, IWT for WT at the IT location, or YWT for WT at the YS location) followed by “D” (for direct mutant allele sequence match) or a numeral giving the quantity of additional mismatches compared to WT sequence introduced into the siRNA. This name then ends with the relative position of the mismatch: c, for center mismatch or s, for shifted mismatch. siRNAs designed with additional mismatches were tested to determine if the changes increased mutant allele specificity by introducing additional nucleotide differences from the WT sequence in cases in which specificity was low (i.e. significant reduction in WT allele expression was observed) [1].

### *In vitro siRNA* Screening at mRNA Level

Since siRNAs promote mRNA degradation, initial evaluation of siRNA-mediated efficacy and specificity was assessed at the mRNA level (Figure 1). For these experiments, HEK293 cells were transfected with one of four different cDNA mini-construct screening plasmids (WT-IT, IT, WT-YS, or YS; see Figure S1C and D) plus 200 pmol of either scrambled (SCR) siRNA or one of the designed allele-specific siRNAs (Figure S1A and B). Total RNA was isolated two days after transfection and semi-quantitative reverse transcriptional polymerase chain reaction (RT-PCR) was used to determine relative knockdown efficacy for each allele separately (Figure 1A and B). After normalization of product to control GAPDH product, the amount of mRNA transcript for each cDNA plasmid was determined and compared to the corresponding level observed following transfection of SCR siRNA (Figure 1C and D).

Figure 1B shows results from a representative RT-PCR experiment illustrating the capacity of this *in vitro* assay to quantify relative mRNA knockdown by different siRNAs. Delivery of siRNAs targeted to WT sequence (IWT-Dc and IWT-Ds) of the IT region of mouse RyR1 (exons 100–105) resulted in an insignificant reduction in WT mRNA (Figure 1C, *left*). Similar results were observed with three siRNA sequences targeted to the IT mutation (IT-Dc, IT-1c, IT-Ds), as evidenced by only a modest reduction compared to SCR control (Figure 1C, *right*). In contrast to results obtained for IT-targeted siRNAs, knockdown efficacy and specificity of three unique siRNAs (YS-Dc, YS-Ds, YS-1s) targeted to the YS region of mouse RyR1 (exons 12–16) resulted in significant mRNA knockdown (Figure 1D). For example, the YS-Dc siRNA reduced YS mRNA by ∼75% (Figure 1D, *right*), but did not significantly knockdown the corresponding WT mRNA (Figure 1D, *left*). Similarly, WT mRNA in this region was markedly reduced only by a siRNA directed against the WT sequence (YWT-Ds), indicating significant siRNA specificity (Figure 1D, *left*). These results indicate that while IT-targeting siRNAs exhibited only marginal knockdown in this assay, the YS-targeted siRNAs exhibited relatively robust and selective mutant-allele mRNA silencing.

### *In vitro siRNA* Screening at Protein Level

**Figure 2:**
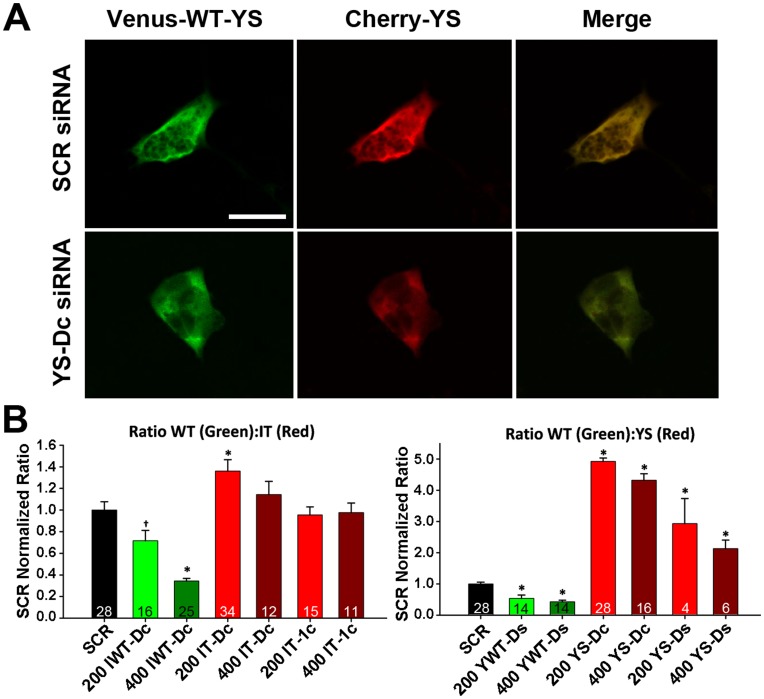
siRNA Screening Results at the Protein Level. Quantification of siRNA knockdown from the ratio of venus:cherry fluorescence intensity evaluated via confocal microscopy. A) Representative venus (*left*), cherry (*middle*), and merged (*right*) images from HEK293 cells co-expressing venus-tagged WT-YS (*left*) and cherry-tagged YS following transfection of scrambled (*top*) or YS-Dc (*bottom*) siRNAs. B) Average (±SEM) siRNA-mediated mRNA knockdown following transfection of either 200 or 400 pmol siRNAs. Venus:cherry ratio was normalized to scrambled control (SCR) for WT-IT versus IT-targeted siRNAs (*left*) and WT-YS vs. YS siRNAs (*right*). * p<0.01 or † p<0.05 vs SCR control. Number of experiments is given at the bottom of each bar in B.

We next extended *in vitro* siRNA screening to the protein level using ratiometric confocal fluorescence microscopy of tagged mini-constructs (Figure 2). Figure 2A shows representative images of green and red fluorescence for a HEK293 cell co-transfected with cDNAs encoding venus-tagged WT and cherry-tagged YS mini-constructs together with either SCR (*top*) or YS-Dc (*bottom*) siRNA. The YS-Dc siRNA caused a selective reduction in cherry fluorescence, resulting in a more green-tinted merged image. Average (±SEM) results for the YS and IT tagged mini-constructs are shown in Figure 2B. The ratio of WT to mutant (venus:cherry) signal normalized to SCR siRNA control was used as an indicator of relative siRNA allele specificity. For the siRNAs targeted to the IT region (Figure 2B, *left*), WT-targeted siRNA (IWT-Dc) showed a modest, but significant, concentration-dependent reduction in WT:mutant ratio, indicating preferential silencing of the WT screening construct. Similarly, IT-directed siRNAs exhibited the opposite effect, with the IT-Dc siRNA significantly increasing WT:mutant ratio. siRNAs designed against the YS region (Figure 2B, *right*) exhibited a similar pattern as IT-directed siRNAs, though with ∼5-fold greater efficacy in target knockdown, consistent with the RT-PCR results shown in Figure 1. In most cases, the 200 pmol concentration of siRNA was equally or more effective in knockdown than the 400 pmol concentration, indicating that siRNA levels above 200 pmol offered no additional benefit. In addition, siRNAs with a direct (D) and centrally located (c) mismatch were most effective and selective for knockdown of both mutant RyR1 mRNA (Figure 1C and D) and protein (Figure 2B). IT-Dc and YS-Dc siRNAs were identified as being the most efficacious and selective for ASGS in the *in vitro* assays, and therefore, were used in the *in vivo* rescue studies below.

### Efficient Widespread Delivery of siRNAs into Hind Footpads of 4–6 Month Old Mice

Targeted widespread delivery of siRNAs into cells of interest is one of the most challenging hurdles to overcome for ASGS. To test the efficacy and specificity of identified siRNAs for ASGS in YS/+ and IT/+ knock-in mice, siRNAs were electroporated into hind footpads of adult mice (see Materials and Methods for details). Using a Cy3-labled siRNA to assess delivery, this approach resulted in introduction of siRNA into >95% of FDB muscle fibers (Figure S2), similar to that observed previously for fluorescently-tagged morpholinos [5]. Though all functional assays were conducted in FDB fibers, comparable delivery was also observed in adjacent interosseous muscles, indicating that this approach provides efficient delivery of siRNAs across all major muscles of the electroporated footpad.

### Assay of Functional Rescue in IT/+ Knock-In Mice

**Figure 3:**
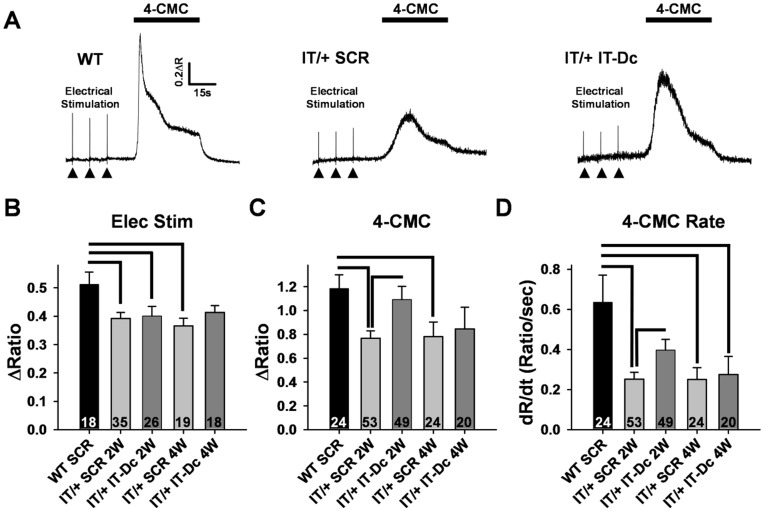
siRNA-mediated Rescue of RyR1 function in FDB fibers from IT/+ knock-in mice. A) Representative indo-1 ratio traces from FDB fibers from a WT mouse treated for two weeks with control scrambled (SCR) siRNA (*left*), a IT/+ mouse treated with SCR siRNA (*middle*), and a IT/+ mouse treated with IT-Dc siRNA (*right*) during electrical stimulation (arrowheads) and application of 500 µM 4-CMC (black bar). B) Average (±SEM) peak magnitude of electrically-evoked Ca^2+^ release in FDB fibers from WT and IT/+ knock-in mice treated with either SCR or IT-Dc siRNA for two (2 W) or four weeks (4 W) as indicated. C) Average (±SEM) peak magnitude and rate (D) of 500 µM 4-CMC-induced Ca^2+^ release in FDB fibers from WT and IT/+ knock-in mice treated with SCR or IT-Dc siRNA for either two (2 W) or four weeks (4 W) as indicated. Statistically significant differences (p<0.05) from WT SCR are indicated with brackets; number of fibers evaluated is given at the bottom of each bar in B-D. Two identically treated animals were used for each experimental condition and a similar number of fibers were used from each of the two animals.

FDB fibers from IT/+ knock-in mice exhibit a significantly reduced magnitude [11], [13] and maximal rate of SR Ca^2+^ release in response to electrical stimulation and exposure to 500 µM 4-cholor-m-cresol (4-CMC), an RyR1 activator [11]. Therefore, we used these parameters as a readout of RyR1 function 2–4 weeks following electroporation of 200 pmol of either scrambled (SCR) control or targeted (IT-Dc) siRNAs into hind footpads of IT/+ mice. Treatment of IT/+ mice for two weeks with 200 pmol of IT-Dc siRNA, one injection per week, resulted in a modest but statistically significant functional rescue of deficits in the peak magnitude (78% rescue, Figure 3A and C) and maximum rate (38% rescue, Figure 3A and D) of 4-CMC-induced Ca^2+^ release. Similar results were also observed following 4 consecutive weeks of treatment (Figure 3B–D). This partial rescue of 4-CMC responsiveness occurred in the absence of a statistically significant effect on maximal electrically-evoked single twitch Ca^2+^ transients (Figure 3 A and B).

### Assay of Functional Rescue in YS/+ Knock-In Mice

**Figure 4:**
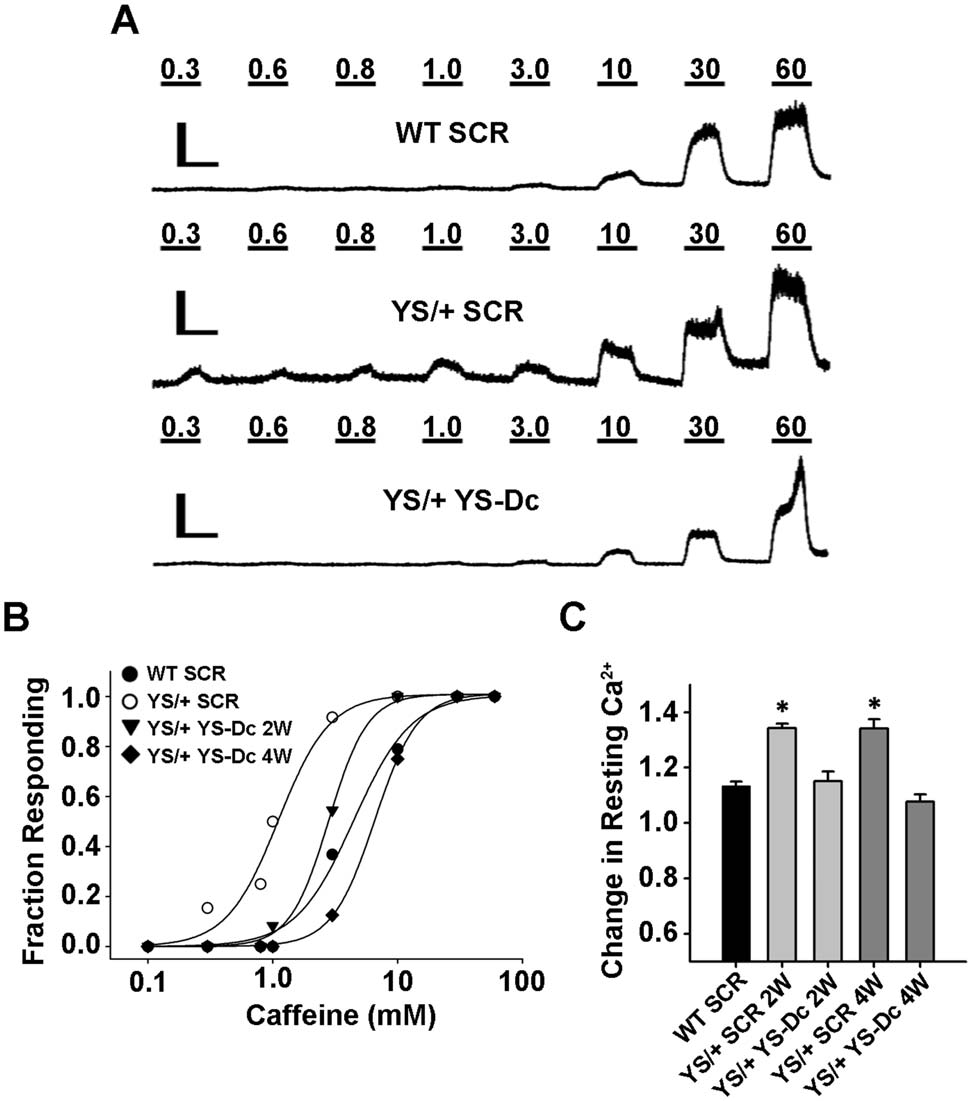
siRNA-mediated Rescue of RyR1 Function in FDB fibers from YS/+ knock-in mice. A) Representative traces of caffeine concentration-responses from FDB fibers obtained from a WT mouse treated for two weeks with SCR siRNA (*top*), a YS/+ mouse treated with SCR siRNA (*middle*), and a YS/+ mouse treated with YS-Dc siRNA (*bottom*). Scale bars represent 30s (*horizontal bar*) and 0.5ΔΔR (*vertical bar*). B) Caffeine-concentration response curves for FDB fibers from WT mice treated with SCR siRNA (*filled circles*, n = 19, EC_50_: 4.44 mM, h: 1.88), FDB fibers from YS/+ mice treated with SCR siRNA (*open circles*, n = 13, EC_50_: 1.11 mM, h: 2.11), and FDB fibers from YS/+ mice treated with YS-Dc siRNA for either 2 weeks (*filled triangles*, n = 13, EC_50_: 2.81 mM, h: 2.80) or 4 weeks (*filled diamonds*, n = 8, EC_50_: 6.57 mM, h: 2.53). For clarity, data for FDB fibers from WT mice treated with SCR siRNA for 4 weeks are similar to the 2 week data, and therefore are not included. C) Fractional change in resting fura-2 ratio upon temperature challenge to 32°C (R_32_/R_25_) in fibers from WT mice treated with SCR siRNA for 2 weeks and YS/+ mice treated for 2 (2 W) or 4 weeks (4 W) with either SCR or YS-Dc siRNA. The number of experiments for each condition is as follows in the format 25^o^C/32^o^C: WT SCR: 160/152; YS/+ SCR 2 W: 104/117; YS/+ YS-Dc 2 W: 39/30; YS/+ SCR 4 W: 104/98; YS/+ YS-Dc 4W: 107/101. * p<0.01. Two identically treated animals were used for each experimental condition and a similar number of fibers were used from each of the two animals.

FDB fibers from YS/+ knock-in mice exhibit an increased sensitivity to activation by both physiological (membrane depolarization) and pharmacological (caffeine) triggers. In addition, FDB fibers from YS/+ mice exhibit a marked temperature-dependent increase in resting Ca^2+^ compared to that observed for FDB fibers from WT mice [20], [21]. Therefore, we utilized the caffeine sensitivity of RyR1 Ca^2+^ release and temperature-dependent changes in resting Ca^2+^ in FDB fibers from YS/+ mice as a readout for the degree of rescue of RyR1 function following footpad electroporation of either scrambled (SCR) control or targeted (YS-Dc) siRNAs. We found that the magnitude of Ca^2+^ release in response to a threshold concentration of caffeine (3 mM) in FDB fibers from YS/+ mice treated for YS-Dc (Peak ΔRatio: 0.14±0.01 and 0.09±0.02 for 2 and 4 week treatment, respectively) were significantly (p<0.01) reduced compared to that of FDB fibers from control SCR siRNA-treated YS/+ mice (Peak ΔRatio: 0.27±0.03 and 0.29±0.03 for 2 and 4 week treatment, respectively). Moreover, the response of FDB fibers from YS/+ mice treated with YS-Dc for either 2 or 4 weeks to 3 mM caffeine was not significantly different from that of fibers from WT mice treated with SCR siRNA (Peak ΔRatio = 0.10±0.02). These results indicate that short-term YS-Dc treatment normalized the enhanced caffeine-responsiveness of FDB fibers from YS/+ mice. Consistent with these results, compared to that of FDB fibers from WT and YS/+ mice treated with SCR siRNA (EC_50_: 1.1 mM and 4.4 mM, respectively), the sensitivity of caffeine-induced Ca^2+^ release was reduced in FDB fibers from YS/+ mice following treatment for 2 weeks (EC_50_: 2.8 mM) and 4 weeks (EC_50_: 6.6 mM) with YS-Dc siRNA (Figure 4B). In addition, the enhanced temperature-dependent increase in resting Ca^2+^ observed in FDB fibers from YS/+ mice was normalized to WT levels following both two and four weeks of treatment with YS-Dc siRNA (Figure 4C).

### Confirmation of *In Vivo* Allele-Specific mRNA Knockdown by qRT-PCR

**Figure 5:**
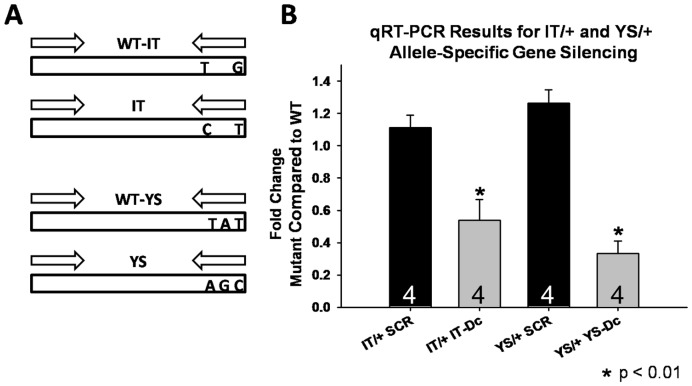
Effect of siRNA Treatment on WT and Mutant Allele Frequency Assessed by qRT-PCR. A) Schematic diagram depicting the allele-specific PCR primer design used to determine fractional WT and mutant allele expression. The IT (*top*) and YS (*bottom*) primer sets amplify 147 and 159 bp PCR products, respectively. Each primer set includes an upstream primer common for both the WT and mutant alleles and a downstream primer designed to specifically anneal to only the WT or mutant template. B) The result of normalized ΔΔCt analysis comparing the fractional contribution of mutant to WT allele frequency (mutant/WT) such that an equal expression is reflected in a value of 1.0 and an increase in fractional WT allele frequency reflected in values <1.0. IT/+ mice were treated for two weeks with either SCR or IT-Dc siRNA. YS/+ mice were treated for two weeks with either SCR or YS-Dc siRNA. A total of n = 4 FDB muscles were used for all conditions. ^*^ p<0.01.

In order to confirm that the functional rescue observed in IT/+ and YS/+ muscle fibers shown in Figures 3 and 4 correlated with preferential knockdown of the mutant RyR1 allele, qRT-PCR was used to quantify relative WT to mutant allele frequency following siRNA treatment (Figure 5). For these experiments, IT/+ and YS/+ mice were treated for two weeks with either SCR or mutant allele-targeted siRNA (IT-Dc or YS-Dc, respectively; Figure 5A). ΔΔCt analysis was used to indicate significant shifts in relative expression levels of WT and mutant alleles in electroporated muscles following treatment. Significant preferential silencing of the mutant alleles was observed following treatment with IT-Dc in IT/+ mice and YS-Dc in YS/+ mice (Figure 5B). Moreover, the degree of relative mutant allele-specific silencing was greater for YS-Dc in YS/+ mice than IT-Dc in IT/+ mice, consistent with the stronger degree of ASGS for YS-Dc observed *in vitro* and the greater functional rescue achieved in YS/+ mice. Similar results were obtained using an alternate approach in which relative WT and mutant allele frequency was determined using digestion of PCR products with allele specific AgeI (for IT) or BlpI (for YS) restriction enzymes (see Materials and Methods for details).

## Discussion

The eukaryotic RNAi system is firmly established as an incredibly powerful tool in research laboratories and its application is now rapidly expanding within the realm of RNA therapeutics [1], [22]–[24]. We developed *in vitro* mRNA- and protein-based assays to screen multiple siRNAs for efficient and allele-specific silencing of RyR1 mini-constructs containing previously identified autosomal dominant human disease mutations. The most promising siRNAs identified in these screens were then evaluated for rescue of RyR1 function following *in vivo* delivery in established autosomal dominant RyR1 knock-in mouse models of MH (Ryr1^Y524S/+^) and CCD (Ryr1^I4895T/+^). The results demonstrate that the degree of siRNA-mediated rescue of RyR1 function closely mirrors the ability of the siRNA treatment to increase fractional WT RyR1 expression by preferentially suppressing expression of the mutant RyR1 allele. These findings provide “proof of principle” for ASGS approaches as a potential future therapeutic intervention for RyR1-linked, and possibly other, autosomal dominantly inherited myopathies.

### siRNA Design Limitations for Autosomal Dominant Mutations

We show that successful application of ASGS for therapeutic benefit in a RyR1-related myopathy requires the siRNA to be capable of preferentially silencing the mutant allele. Thus, siRNA design for this purpose necessitates properly targeting the site of the disease mutation and/or using an identically segregated polymorphism. Beyond this limitation, the most important sequence region for binding energy and control of off-target effects is bases 2–8 of the antisense strand [25], [26]. This, combined with efficient cleavage of mRNA at centrally located nucleotides [26], leaves two rational options for optimized siRNA design: centrally located mismatch and seed region mismatch. Within these basic parameters, we screened several of the best possible siRNAs according to an accepted rational siRNA design algorithm [16].

Although a similar siRNA design strategy was used for both YS/+ and IT/+ mice, significantly greater ASGS and RyR1 functional rescue was observed for the YS-directed siRNAs (e.g. YS-Dc). The greater efficacy of the YS-directed siRNAs could reflect the greater number of nucleotide mismatches between WT and mutant sequences (the engineered YS knock-in mutation includes three nucleotide differences while the IT mutation has two), differences in steric obstruction of the target strand due to distinct mRNA secondary structures between the two target regions, or a combination of these two factors. Regardless of the reason for this difference in efficacy, our studies demonstrate that the relative degree of siRNA-mediated increase in fractional WT allele expression is a key factor in determining functional rescue.

### siRNA Screening: A Reductionist Approach for ASGS

We screened multiple potential siRNAs under both *in vitro* “homozygous” (mRNA analysis) and “heterozygous” (protein analysis) expression conditions in HEK293 cells. This approach enabled streamlining the process of identifying optimal siRNAs for preferential mutant allele knockdown. A key to the successful implementation of this system to screen for RyR1 knockdown was the construction of minimal-sized screening constructs that express a series of RyR1 exons that include and surround the mutation site. This approach enabled a dramatic reduction in the size of the insert from ∼15 kb to less than 1 kb. The creation of RyR1 cDNA mini-constructs increased both ease of molecular manipulation and transfection efficiency and enabled far more rapid screening of multiple siRNAs than would otherwise be possible. Analysis of relative protein expression by confocal fluorescence microscopy significantly limits high-throughput, though the approach provides critical confirmation of siRNA specificity at the protein level and under heterozygous expression conditions. Rapid screening of large cell populations via fluorescence-activated cell sorting would enable considerably greater screening throughput at the protein level.

### Functional Rescue in IT/+ and YS/+ Mice

Our results demonstrate that acute (2–4 weeks) preferential silencing of the mutant allele is able to normalize the primary defects in RyR1 function (e.g. electrically-evoked and ligand-induced Ca^2+^ release, caffeine sensitivity, resting Ca^2+^) in muscle fibers from YS/+ and IT/+. Prominent alterations in muscle ultrastructure are also observed in both YS/+ [9] and IT/+ [12], [13] mice, though these alterations develop slowly over time as the mice age (e.g. over 6–12 months). Thus, an important goal for future studies will be to determine if long-term systemic delivery of the ASGS siRNAs is able to prevent and/or correct the secondary ultrastructural changes in muscle observed in YS/+ and IT/+ mice. Indeed, the MH (YS/+) and CCD (IT/+) RyR1 knock-in mice used in this study provide powerful *in vivo* models to evaluate RyR1 functional rescue by siRNA-directed ASGS. Interestingly, although the two mouse models used here result from both RyR1 gain (YS/+) and loss (IT/+) of function mutations, significant siRNA-mediated functional rescue was observed for both models. These findings provide sound rationale for extending this work to systemic administration of these and other siRNAs in order to assess effects on relative WT:mutant RyR1 expression across multiple muscles groups, as well as the degree to which these changes correlate with correction of enhanced anesthetic/heat hypersensitivity [7] and age-dependent development of unstructured cores [9] in YS/+ knock-in mice, as well as muscle weakness [11], [12] and altered muscle ultrastructure [12], [13] in IT/+ knock-in mice. Such systemic siRNA administration rescue studies should soon be feasible given recent advances in the development of several safe and effective systemic siRNA delivery systems [24].

An unexpected finding of this study was that treatment of IT/+ mice with the IT-Dc siRNA was able to partially restore 4-CMC responses, but not electrically-evoked Ca^2+^ release (Fig. 3). Although a tendency for an increase in the magnitude of electrically-evoked Ca^2+^ release was observed following treatment with IT-Dc for 4 weeks (see Fig. 3B), these data did not reach statistical significance. The reason for this discrepancy is not entirely clear. However, one possibility is that since the IT-Dc siRNA produced only a modest degree of mutant allele-specific silencing (Figs. 2B and 5B), functional rescue may be more easily resolved for the slow and large 4-CMC responses with the high affinity Ca^2+^ indicator indo-1 used in this study. The use of a lower affinity Ca^2+^ dye with considerably faster kinetics (e.g. Mag-fluo-4; see Loy et al., 2010) might provide a more robust means of resolving modest changes in the magnitude and maximum rate of electrically-evoked Ca^2+^ release following treatment with IT-Dc.

### Future Implications

siRNA-based treatments are currently being developed and evaluated in clinical trials for wide range of disorders including macular degeneration, glaucoma, respiratory synical virus infection, pachyonychia congenital, asthma, cancer, hypercholesterolemia, and HIV [2], [24], [27]. Our findings in which altered RyR1 function in FDB fibers of YS/+ and IT/+ knock-in mice can be normalized only two weeks after local *in vivo* delivery of ASGS siRNAs provide “proof-of-principle” support for the extension of these studies to systemic administration, and ultimately, for the future development of similar siRNA-based therapeutic strategies to treat RyR1-linked autosomal dominantly inherited myopathies in humans.

## Supporting Information

Figure S1 **siRNA Design and Screening Constructs.** A-B) siRNAs were designed and described utilizing the following nomenclature for I4895T (A) and Y524S (B) knock-in mice: Genotype-Sequence Match-(D = direct or # of mismatches)Position(c, centered mismatch or s, shifted mismatch). C-D) siRNA screening plasmids constructed by in-frame insertion of the indicated murine WT (*left*) or mutant (*right*) RyR1 exons between venus and a 3xFLAG tag (WT) or cherry and a 3xHA tag (mutants). C) Constructs used for screening WT and IT siRNAs containing RyR1 exons 100–105. D) Constructs used for screening WT and YS siRNAs containing RyR1 exons 12–16.
(DOCX)

Figure S2 **Highly Efficient Delivery of siRNAs into FDB Fibers Following** ***in vivo*** **Electroporation.** A) Representative low magnification wide field transillumination image of FDB fibers isolated from a WT mouse one week after electroporation with a cy3 labeled control siRNA. (B) Fluorescence image of the field in A showing that all fibers exhibit significant red Cy3 fluorescence. C) Representative high magnification transillumination image of four adjacent FDB fibers isolated from a WT mouse 1 week after electroporation with Cy3 labeled control siRNA. D) Confocal image of the field in C showing that all four FDB fibers exhibit significant red Cy3 fluorescence. Red: Cy3 siRNA (543 nm excitation). Blue: Nuclei (Hoechst, 405 nm excitation). Scale bars represent 20 µm for A and B and 5 µm for C and D.
(DOCX)
