# Supplementary figures and images for: Allele-Specific Gene Silencing in Two Mouse Models of Autosomal Dominant Skeletal Myopathy
Source: PLoS One. 2012 Nov 12;7(11):e49757. doi: 10.1371/journal.pone.0049757 (PMC3495761; doi:10.1371/journal.pone.0049757)

**
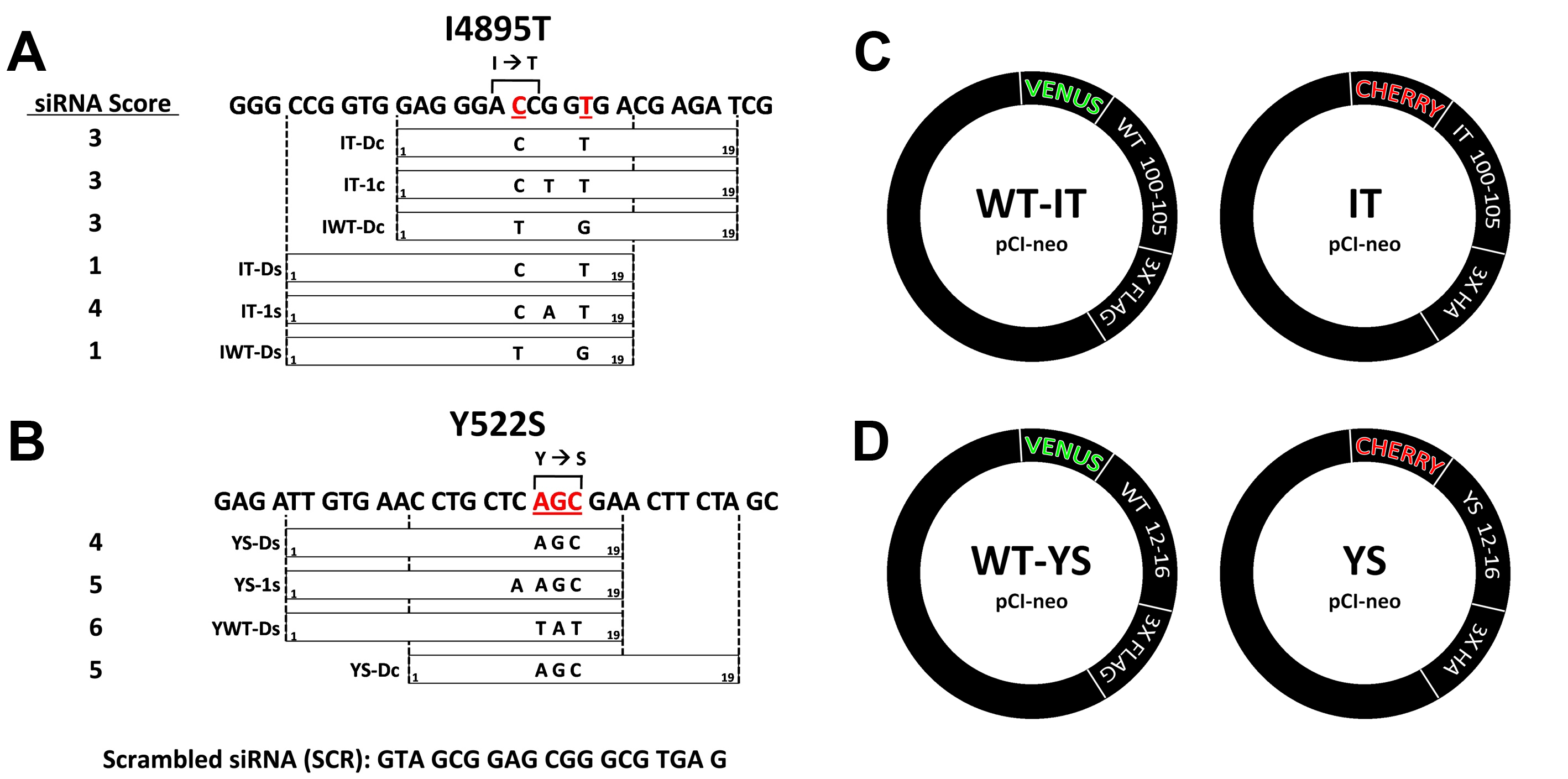
**

Supplement: Figure S1 — siRNA Design and Screening Constructs. A-B) siRNAs were designed and described utilizing the following nomenclature for I4895T (A) and Y524S (B) knock-in mice: Genotype-Sequence Match-(D = direct or # of mismatches)Position(c, centered mismatch or s, shifted mismatch). C-D) siRNA screening plasmids constructed by in-frame insertion of the indicated murine WT (left) or mutant (right) RyR1 exons between venus and a 3xFLAG tag (WT) or cherry and a 3xHA tag (mutants). C) Constructs used for screening WT and IT siRNAs containing RyR1 exons 100–105. D) Constructs used for screening WT and YS siRNAs containing RyR1 exons 12–16. (DOCX) [file pone.0049757.s001.docx]

**
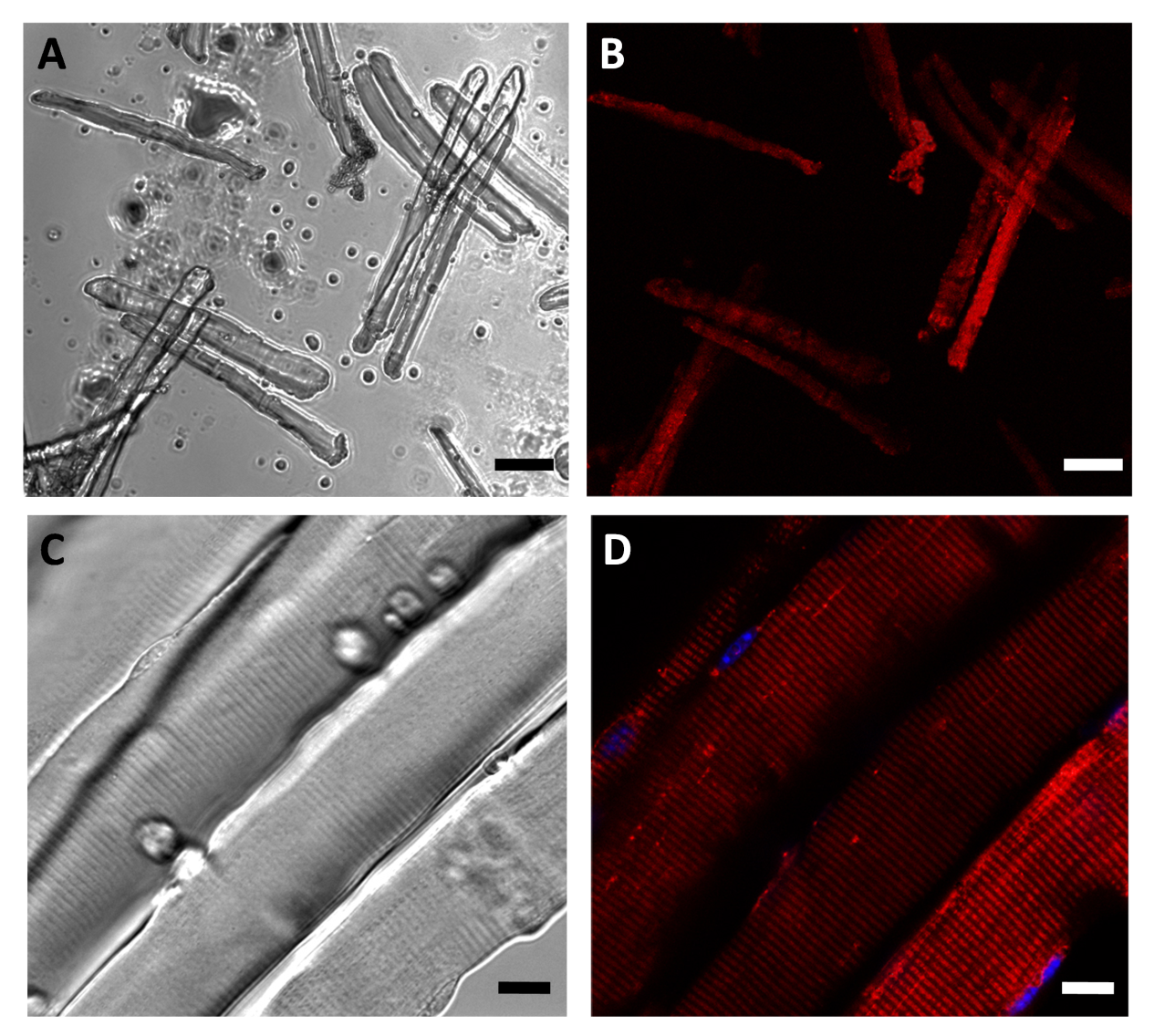
**

Supplement: Figure S2 — Highly Efficient Delivery of siRNAs into FDB Fibers Following in vivo Electroporation. A) Representative low magnification wide field transillumination image of FDB fibers isolated from a WT mouse one week after electroporation with a cy3 labeled control siRNA. (B) Fluorescence image of the field in A showing that all fibers exhibit significant red Cy3 fluorescence. C) Representative high magnification transillumination image of four adjacent FDB fibers isolated from a WT mouse 1 week after electroporation with Cy3 labeled control siRNA. D) Confocal image of the field in C showing that all four FDB fibers exhibit significant red Cy3 fluorescence. Red: Cy3 siRNA (543 nm excitation). Blue: Nuclei (Hoechst, 405 nm excitation). Scale bars represent 20 µm for A and B and 5 µm for C and D. (DOCX) [file pone.0049757.s002.docx]
